# Identification of extracellular nanoparticle subsets by nuclear magnetic resonance†

**DOI:** 10.1039/d1sc01402a

**Published:** 2021-04-29

**Authors:** Md Sharif Ullah, Vladimir V. Zhivonitko, Anatoliy Samoylenko, Artem Zhyvolozhnyi, Sirja Viitala, Santeri Kankaanpää, Sanna Komulainen, Leif Schröder, Seppo J. Vainio, Ville-Veikko Telkki

**Affiliations:** NMR Research Unit, University of Oulu Finland ville-veikko.telkki@oulu.fi Vladimir.zhivonitko@oulu.fi; Laboratory of Developmental Biology, Infotech Oulu, Oulu Center for Cell-Matrix Research, Kvantum Institute, Faculty of Biochemistry and Molecular Medicine Oulu Finland Anatoliy.Samoylenko@oulu.fi; Production Systems, Natural Resources Institute Finland (Luke) Jokioinen Finland; Molecular Imaging, Leibniz-Forschungsinstitut für Molekulare Pharmakologie (FMP) Berlin Germany lschroeder@fmp-berlin.de; Division of Translational Molecular Imaging, German Cancer Research Center (DKFZ) Heidelberg Germany leif.schroeder@dkfz-heidelberg.de

## Abstract

Exosomes are a subset of secreted lipid envelope-encapsulated extracellular vesicles (EVs) of 50–150 nm diameter that can transfer cargo from donor to acceptor cells. In the current purification protocols of exosomes, many smaller and larger nanoparticles such as lipoproteins, exomers and microvesicles are typically co-isolated as well. Particle size distribution is one important characteristics of EV samples, as it reflects the cellular origin of EVs and the purity of the isolation. However, most of the physicochemical analytical methods today cannot illustrate the smallest exosomes and other small particles like the exomers. Here, we demonstrate that diffusion ordered spectroscopy (DOSY) nuclear magnetic resonance (NMR) method enables the determination of a very broad distribution of extracellular nanoparticles, ranging from 1 to 500 nm. The range covers sizes of all particles included in EV samples after isolation. The method is non-invasive, as it does not require any labelling or other chemical modification. We investigated EVs secreted from milk as well as embryonic kidney and renal carcinoma cells. Western blot analysis and immuno-electron microscopy confirmed expression of exosomal markers such as ALIX, TSG101, CD81, CD9, and CD63 in the EV samples. In addition to the larger particles observed by nanoparticle tracking analysis (NTA) in the range of 70–500 nm, the DOSY distributions include a significant number of smaller particles in the range of 10–70 nm, which are visible also in transmission electron microscopy images but invisible in NTA. Furthermore, we demonstrate that hyperpolarized chemical exchange saturation transfer (Hyper-CEST) with ^129^Xe NMR indicates also the existence of smaller and larger nanoparticles in the EV samples, providing also additional support for DOSY results. The method implies also that the Xe exchange is significantly faster in the EV pool than in the lipoprotein/exomer pool.

## Abstract 2

Diffusion and xenon NMR based methods to determine a very broad range of sizes and sub-sets of extracellular vesicles.

## Graphical abstract

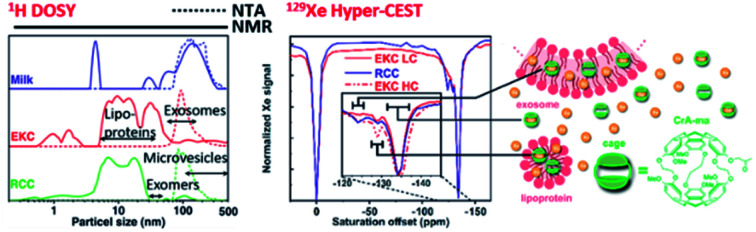

## Introduction

Exosomes are nanosized (50–150 nm) vesicles widely secreted by cells.^1,2^ Over the last few years, these extracellular vesicles (EVs) have gained attention for their abundance in biological fluids^3–5^ and their roles in multiple physiological processes.^6^ Their physicochemical characterization is thus of utmost importance. Because exosomes transfer compounds such as lipids, proteins, metabolites, DNA, and RNA to target cells, they are also called signalosomes.^7–9^ They are also promising vesicles for drug delivery due to their low toxicity, biological membrane permeability, and low immunogenicity.^9^ Lipids and proteins available at the surface of exosomes can act as biomarkers, specifying their secreting cell. Hence, exosomes can be potentially used for the diagnosis of diseases like cancers.^10^

The identification of EV subpopulations as well as their properties is not trivial due to their small size and the existence of many co-isolated nanoparticles in the solution. Several techniques such as transmission electron microscopy (TEM),^11^ flow cytometry, resistive pulse sensing (RPS), and nanoparticle tracking analysis (NTA)^12^ have been used for determining the concentration and particle size distribution of EVs. Due to biological sample heterogeneity, the results of different techniques may vary significantly.^13^ Currently, NTA is probably the most popular characterization method. It detects Brownian motion of nanoparticles in liquid suspensions by light scattering microscopy to determine particle size distributions.^14^ However, NTA has a limited ability to observe small nanoparticles.

Nuclear magnetic resonance (NMR) spectroscopy provides versatile chemical, dynamic and spatial information of molecules in solution non-invasively.^15^ For example, it allows the determination of 3D structures of biological macromolecules in solution,^16^ as well as conformations and functions of proteins in living cells.^17^ NMR is also one of the few methods for quantification of molecular self-diffusion non-invasively without tracers.^18^ As the translational diffusion coefficient reflects the sizes and shapes of molecules or complexes, diffusion ordered spectroscopy (DOSY) is a powerful tool for analyzing complex mixtures.^19^ For obtaining complementary insights, ^129^Xe (26.4% natural abundance) is an excellent NMR probe, because it is chemically inert, and its chemical shift is extremely sensitive to its local environment.^20–23^ In addition, its NMR sensitivity can be increased up to five orders of magnitude by spin-exchange optical pumping (SEOP) as a well-established hyperpolarization technique. Combination of hyperpolarization and chemical exchange saturation transfer (CEST)^24^ techniques (Hyper-CEST) enables very high-sensitivity, background-free molecular detection of biological systems.^25–29^

So far, the application of NMR spectroscopy in EV research has been scarce. Shao *et al.* developed a microfluidic system for on-chip NMR (μNMR) detection of circulating microvesicles.^30^ The EVs were labelled with magnetic nanoparticles, which enhanced *T*_2_ relaxation, and *T*_2_ decay rate enabled the quantification of EV concentration.

Here, we exploit, for the first time, ^1^H DOSY and ^129^Xe Hyper-CEST in the characterization of EVs. We demonstrate that ^1^H DOSY can be used for the investigation of dynamics, size distribution and sub-populations of EVs extracted from various sources. We show that the technique also enables the observation of small extracellular nanoparticles, which are invisible in standard NTA analysis. Contrary to the μNMR, the DOSY analysis does not require magnetic nanoparticle labeling, but it is non-invasive. Furthermore, we show that Hyper-CEST method provides an improved specificity for exosomes and extracellular nanoparticles of different sizes. This technique offers exceptional sensitivity and specificity. The spin label is introduced by simple gas dispersion and does not require elaborate isotope labelling like in other hyperpolarization approaches. Compared to the conventional ^129^Xe NMR spectroscopy, it identifies additional signals, and the analysis supports the existence of at least two subpopulations of the nanoparticles.

## Materials and methods

### EVs purification

Mouse renal adenocarcinoma-derived Renca cells (ATCC® CRL-2947™) and UB stalk cells, derived from embryonic kidney ureteric bud (a gift from Satu Kuure laboratory, Helsinki University), were cultured in DMEM medium supplemented with 10% fetal bovine serum (FBS), 100 U ml^−1^ penicillin and 100 μg ml^−1^ streptomycin at 37 °C in 5% CO_2_. Cell culture medium was changed to the one without FBS 24 h before EV isolation. After that, the medium was collected for EV isolation. EVs were purified from cell culture media by the combination of sequential ultracentrifugation and size-exclusion chromatography with Exo-spin™ columns (Cell Guidance Systems Ltd). In brief, collected medium was centrifuged at 5000 rpm for 30 min, and the obtained supernatant was concentrated using Centricon Plus-70 filter units (Merck Millipore, cut-off 100k) for 20 to 40 minutes and diluted with PBS to the total volume of 10 ml. The samples were centrifuged at 100 000*g*, 4 °C (Sorvall TH-641 rotor) for 18 hours and pellets diluted in 200 μl PBS. While several samples (labelled “no exospin”) were used for DOSY NMR analysis after this step, others were further purified using Exo-spin™ kit (EXO3) according to the manufacturer's protocol.

EVs were purified from cow's milk by differential centrifugation combined with sequential filtering. Milk samples were collected in the research barn of Natural Resources Institute Finland (Jokioinen, Finland) and kept in +4 °C until EV purification. Milk samples were centrifuged twice at 3000*g* and +4 °C for 15 min (JA-17 rotor, Beckman Coulter) to separate milk fat and to pellet intact cells and cellular debris. The middle layer was then further centrifuged at 21 500*g* and +4 °C for 30 min to pellet milk caseins and larger vesicles. The step was repeated for 60 min at 21 500*g* and +4 °C. Resulting solution was filtered through hydrophilic syringe filters of 0.8 μm (Minisart®, Santorius, Göttingen, Germany), 0.45 μm and 0.22 μm (LLG labware, Meckenheim, Germany) to remove larger particles such as milk fat globules. EVs were then sedimented from the filtrate by ultracentrifugation (Type 50.2 Ti rotor, Beckman Coulter L8-80M) at 100 000*g* and +10 °C for 90 min. The resulting gel-like and transparent pellet was resuspended to 2 ml of phosphate buffered saline (137 mM NaCl, 2.7 mM KCl, 10 mM Na_2_HPO_4_, 1.8 mM KH_2_PO_4_, pH 7.4 Cold Spring Harbor Protocols) and further purified by ultracentrifugation at 100 000*g* and +10 °C for another 90 min. Resulting clear pellet was dissolved to phosphate buffered saline overnight in +4 °C under mild rocking. The samples were stored in aliquots at −80 °C.

In total, two EV samples were extracted from milk, five from embryonic kidney cells (EKC), and four from renal carcinoma cells (RCC). The samples are named accordingly as Milk 1–2, EKC 1–5, and RCC 1–4.

### ^1^H DOSY NMR analysis

For the DOSY NMR analysis, 30 μl of a stock EV solution was premixed with 6 μl of deuterium oxide (D_2_O) and transferred into a 1.7 mm NMR tube. The ^1^H DOSY NMR analysis was performed with a Bruker Avance 500 MHz spectrometer, using a 1.7 mm TXI micro cryoprobe with a *z* gradient and a pulsed-gradient stimulated-echo (PGSTE) pulse sequence with bipolar gradients. The DOSY decay curves were measured with 32 magnetic field gradient values that increased linearly from 2.89 G cm^−1^ up to maximally 45.74 G cm^−1^. The measurements were performed at slightly lowered temperature (280 K) to avoid the decomposition of samples during the experiments. The diffusion time *Δ* was 120 ms, the effective gradient pulse duration *δ* was 24 ms, the maximum *b*-value was 9.65 × 10^11^ s m^−2^ and the recycling delay was 2 s. Due to the low concentration of EVs, signal-to-noise ratio in the spectra was low, and 1024 scans were accumulated in order to get reasonably good signal-to-noise ratio in the spectra. Therefore, the experiment time was long, approximately 50 hours, but the stability of the sample over the measurement period is not a problem as EVs are rather stable at low temperatures.^31^ The experiment data were processed in Bruker TopSpin 3.6.0 and Origin 2018b. Automatic polynomial fit was used for baseline correction and phasing was done using manual phase correction. The algorithm based on adaptive truncation of matrix decompositions was used for Laplace inversion.^32^ The value of parameter *α* scaling the weight of the Tikhonov regularization was adjusted in a standard way by running the *α* loop.

### ^129^Xe Hyper-CEST analysis

^129^Xe Hyper-CEST experiments were performed on a Bruker Ultra-Shield-Plus 400 MHz NMR spectrometer using a 10 mm double-resonant ^1^H/^129^Xe probe at room temperature. The Hyper-CEST samples included EVs (final concentrations of EKC and RCC 2.13 × 10^10^ and 1.89 × 10^10^ particles per ml, respectively), 20 μM cryptophane-A mono-acid cages (CrA-ma; provided by Kangyuan Jiyi Inc., Shangdong, PRC), and 1 μl of Pluronic L-81 (a block copolymer serving as antifoaming surfactant) in phosphate-buffered saline (PBS). CrA-ma self-embeds into phospholipid membranes^33–35^ and serves as a marker to give transiently bound Xe a distinct chemical shift to discriminate it from free Xe in solution. The total volume of the sample was about 1 ml.

Hyperpolarized ^129^Xe was produced in a continuous flow mode employing a home-build spin-exchange optical pumping (SEOP) polarizer.^36^ A gas mixture including 5% of Xe (plus 10% N_2_ and 85% He) with natural abundance of ^129^Xe was delivered to the sample at the flow rate of 70 standard milliliters per minute (SMLM) at a total pressure of 1.8 bar. Hyperpolarized ^129^Xe was bubbled through the sample solution using four fused silica capillary tubes (350 μm outer diameter). Each acquisition loop started with a 3 s pre-delay to allow for any solution pushed up the glass wall (from the previous Xe delivery) to settle back into the bulk volume. Xe gas mix delivery started then for 10 s, followed by a 5 s delay for dissipation of remaining foam. This was immediately followed by the Hyper-CEST encoding as follows: the saturation transfer was induced by applying a 2.5 mW saturation pulse (0.605 μT) for 25 s, followed by a Gauss-shaped 90° pulse for selective read-out of the Xe bulk magnetization in water (referenced to 0 ppm). The FID signal was sampled for 0.5 s. Hyper-CEST spectra were measured by varying the saturation offset with a non-uniform frequency list between +80 and −160 ppm. Data in the range −122 … −140 ppm was sampled in 1 ppm steps. For each dataset, four initial Xe deliveries and acquisitions with 200 ppm saturation offsets and three final ones at −200 ppm offset were included for allowing (a) a stable Xe level to begin with and (b) to include far off-resonant data points to define the reference signal of unsaturated Xe in solution. Altogether, 60 points were collected and the total experiment time was about 45 min. A *z*-spectrum at higher resolution was taken with non-uniform sampling of saturation offsets between −105 ppm and −160 ppm (53 offsets). The high-resolution range −122 … −138 ppm was sampled in increments of 0.5 ppm. Including the far off-resonant scans, 60 offsets were again included in these data sets.

## Results and discussion

### DOSY and NTA particle size distributions

**Fig. 1:**
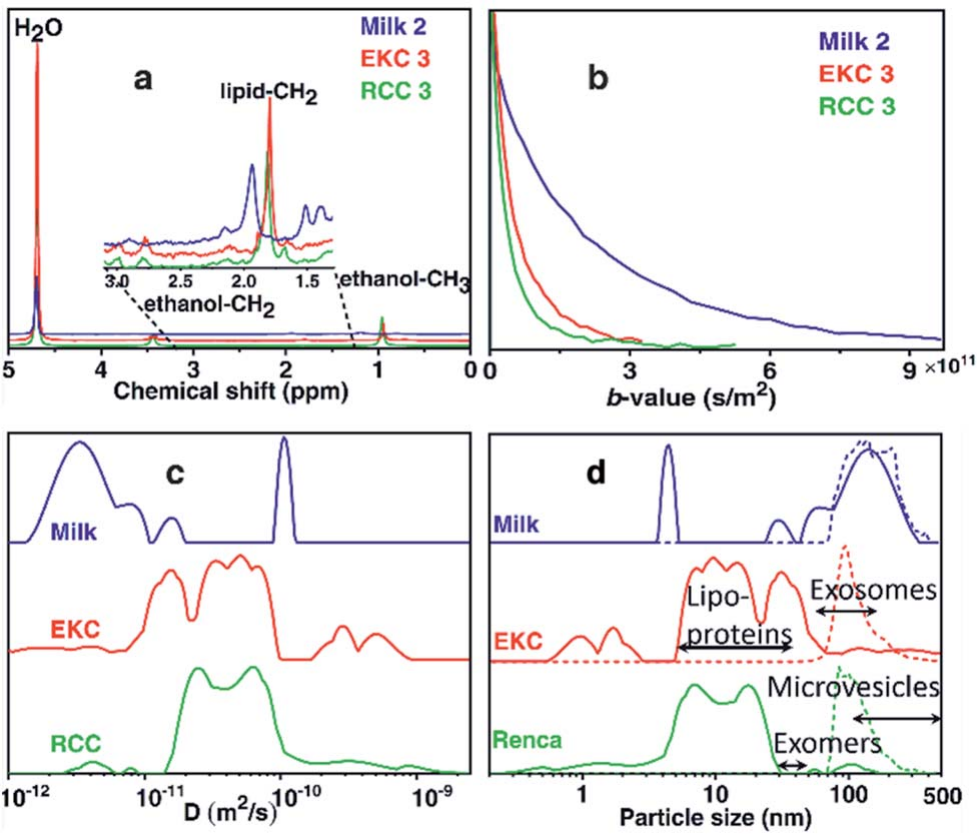
DOSY NMR analysis of EV samples. (a) ^1^H DOSY spectra (corresponding to the smallest gradient value) of selected milk, EKC and RCC EV samples. (b) DOSY decay curves of the lipid CH_2_ signals. (c) Sums of diffusion coefficient distributions of all milk, EKC and RCC samples. (d) Particle size (diameter) distributions derived from the diffusion coefficient distributions by Stokes–Einstein equation (solid lines). For comparison, corresponding NTA distributions are shown with dashed lines.

Fig. 1a shows the ^1^H spectra of milk, EKC, and RCC samples corresponding to the smallest gradient value in the DOSY experiments. In addition to the signals of water (4.7 ppm) and ethanol (0.9 and 3.4 ppm, a component of the buffer, in which Exo-spin™ columns were supplied), the spectra include small signals in the region of 1.0–3.5 ppm which were interpreted to arise from exosomes as well as other co-isolated EVs, particles and molecules. The dominant EV signal between 1.8 and 2.0 ppm was interpreted to originate from lipid CH_2_ groups. No signals were observed in the aromatic region (around 7 ppm).

The DOSY decay curves of the dominant EV signals of representative milk, EKC and RCC samples are shown in Fig. 1b. The milk signal is decaying much slower than the EKC and RCC signals, indicating larger average particle size in the former case. The decay curves were converted into diffusion coefficient (*D*) distributions by the inverse Laplace transform (ILT).^32,37,38^Fig. 1c shows the sums of all *D* distributions of each sample type (the distributions of each individual experiments are discussed in section “DOSY and NTA of individual samples” below). The observed diffusion coefficients vary between 10^−12^ and 10^−9^ m^2^ s^−1^, and the smaller values represent larger complexes. There are many peaks in each distribution, indicating a broad particle size distribution. The maximum reliably observable *D* is about *D*_max_ = 3 × 10^−10^ m^2^ s^−1^ (an inverse of the minimum *b*-value, see Table S1 in ESI†). The small peaks observed above *D*_max_ are heavily influenced by the noise.

The diffusion coefficient distributions were converted into particle size distributions by using the Stokes–Einstein equation.^39^ This approach should be a reasonable approximation for spherical EVs, which are much larger than the solvent molecules (H_2_O),^40–42^ and the effect of obstruction^43,44^ is assumed to be insignificant due to low concentration of EVs. The resulting particle size distributions (solid lines), along with the NTA distributions (dashed lines), are shown in Fig. 1d. We note that the DOSY and NTA distributions shown here represent slightly different things, as the former depicts the number of spins while the latter represents the number of particles *vs.* size, and therefore the intensities of the distributions are not directly comparable. The DOSY spin number distributions could be converted into the particle number distributions, *e.g.*, by assuming that the number of spins is proportional to the surface area of EVs, but we do not show this conversion as the spin density may be different for different types of particles. The minimum reliably observed particle size in the DOSY experiments is about 1 nm (calculated from *D*_max_). The NTA distributions overlap with the DOSY distributions; both distributions show non-zero intensities around 70–400 nm. However, the DOSY distributions also include a significant number of smaller particles in the range of 1–70 nm, which are not observable with NTA. Overall, the DOSY distributions include particles in a very broad size range of 1–500 nm, which is significantly exceeding the size range of exosomes (50–150 nm). Typically, there are many other particles that are co-isolated together with exosomes such as lipoproteins (HDL, LDL and IDL, 5–35 nm), non-membranous exomers (30–50 nm),^45^ and microvesicles (100–1000 nm),^1^ and, most probably, these particles contribute the observed DOSY distributions as well.

**Fig. 2:**
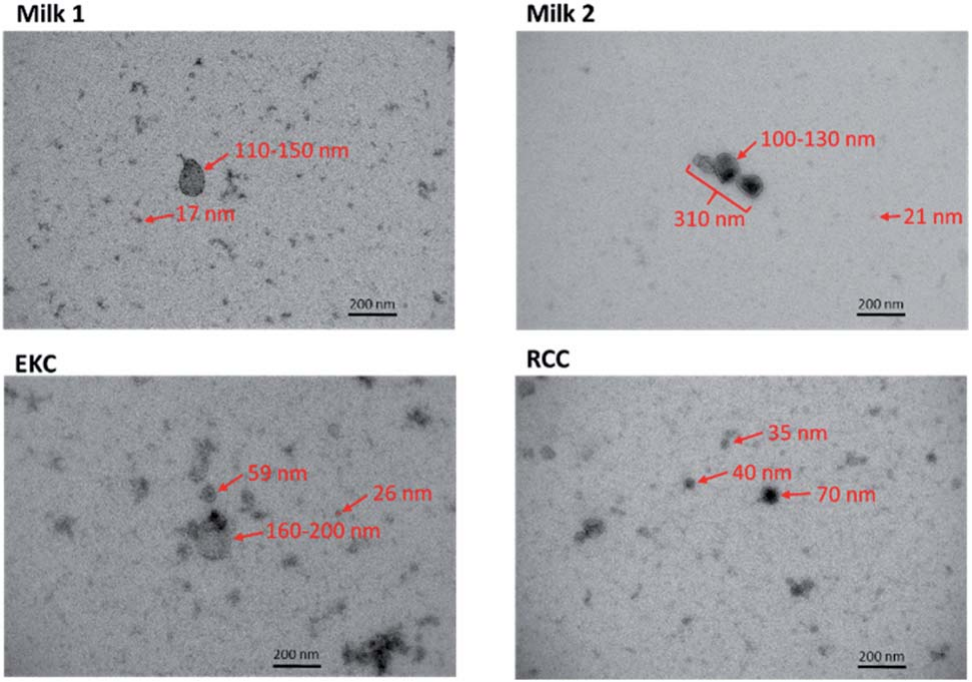
Transmission electron microscopy images of some milk, EKC and RCC EV samples. The estimated diameters of some particles are shown in the images.

The DOSY distribution of the milk samples includes a narrow peak of small particles around 4 nm, which may be associated with small molecular aggregates formed by phospholipids. Furthermore, there is a broad distribution of large particles around 20–350 nm, which may include exosomes, microvesicles, nano-micro-sized fat globules and casein micelles (average size about 150 nm).^46^ The average size of the particles is significantly larger in the milk samples than in the other samples. The NTA distribution shows particles in the range of 70–400 nm and is in good agreement with the DOSY distribution in the size range, in which the method allows the detection of particles. NTA implies also that the mean particle size in the milk samples is larger than in the EKC and RCC samples. The size of the largest particles observed by both DOSY (350 nm) and NTA (400 nm) is larger than the smallest filter size used in EVs purification (220 nm). This may be a consequence of aggregation of EVs, which was observed in TEM images (Fig. 2).

The dominant peaks of the DOSY distributions of the EKC samples lie between 5 and 70 nm, and they may be associated with lipoproteins, exomers, and small exosomes. There is also a peak corresponding to very small particles around 1–2 nm, which may arise from free (lipid) molecules or very small molecular aggregates. Furthermore, there are small intensity peaks around 70–500 nm, which are overlapping with the NTA distribution, and may correspond to larger exosomes as well as microvesicles. The smaller intensity in the DOSY distribution is partially a consequence of different scaling of the DOSY and NTA distributions, because the distributions were normalized based on the maximum intensity, and in the DOSY distributions the maxima are in the region, which is invisible by NTA.

The DOSY particle size distribution of RCC samples resembles that of EKC samples, but the mean particle size is smaller. The dominant peaks lie between 4 and 30 nm and may originate from small molecular aggregates and lipoproteins. The signal around 30–70 nm is much smaller than in the EKC samples, implying a smaller relative amount of exomers and small exosomes. There is a small signal of larger particles around 70–200 nm, which is partially overlapping with the NTA distribution, and may arise from larger exosomes and microvesicles.

### TEM images

TEM images of some milk, EKC and RCC samples in Fig. 2 support the DOSY NMR observations. The images include many particles with different sizes. The largest particles are 150–200 nm in diameter. The Milk 2 image includes an aggregate of three particles with the length of about 300 nm. There are large number of particles with their diameter below the detection limit of the NTA analysis (∼70 nm). The milk images include large (100–300 nm) and small (below 20 nm) particles, which supports the bimodal distribution observed by DOSY NMR. The EKC image shows a broad and continuous particle size distribution from a few to 200 nm, which is also in agreement with DOSY NMR. The particles visible in RCC images are slightly smaller than those in EKC images, being again in agreement with DOSY NMR. The TEM images include also smaller dark dots, which may represent the smallest particles in the DOSY distributions, but which are close to the practical resolution limit of the images.

### DOSY and NTA of individual samples

**Fig. 3:**
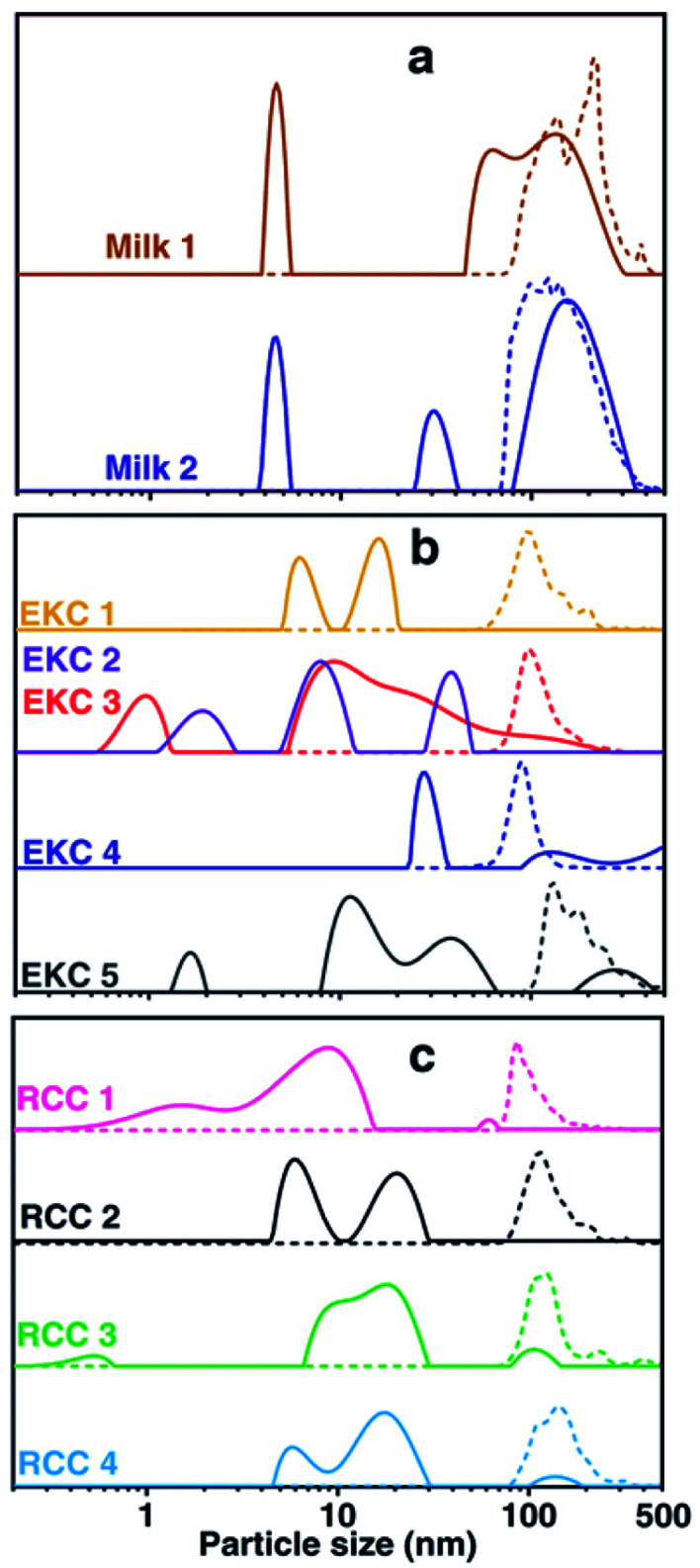
DOSY and NTA of individual samples. Size (diameter) distribution of all milk (a), EKC (b) and RCC (c) samples measured by DOSY NMR (solid lines) and NTA (dashed lines). EKC 2 and 3 DOSY samples were taken from the same sample batch, and only one NTA was measured for the batch.

The DOSY and NTA particle size distributions for each individual sample are shown in Fig. 3. These distributions were summed together to have the distributions shown in Fig. 1d. The DOSY distribution within each sample type (milk, EKC and RCC) varies a lot. This reflects partially the differences between samples, but, most probably, artefacts of the applied evaluation also play a significant role. It is well known that the inverse Laplace transform has a limited stability, and some of the small signals and features in the distributions may be a consequence of the noise in the measurement data.^47,48^ In order to have a reliable multicomponent DOSY diffusion coefficient distribution, the signal-to-noise (SNR) ratio in the DOSY decay curve should be over 100.^49,50^ However, SNR in these measurements was below 100 (Fig. 1b). Therefore, the positions and intensities of the peaks are affected by noise, but the mean reflects well the average particle size. On the other hand, as the distribution shown in Fig. 1d are sums of distributions of 2–7 samples, they should reliably reflect the average particle size distributions of the samples.

According to the NTA, the particle size distributions of different milk and RCC samples are quite similar. However, EKC 5 sample seems to include larger particles than the other EKC samples.

### ^129^Xe Hyper-CEST analysis

**Fig. 4:**
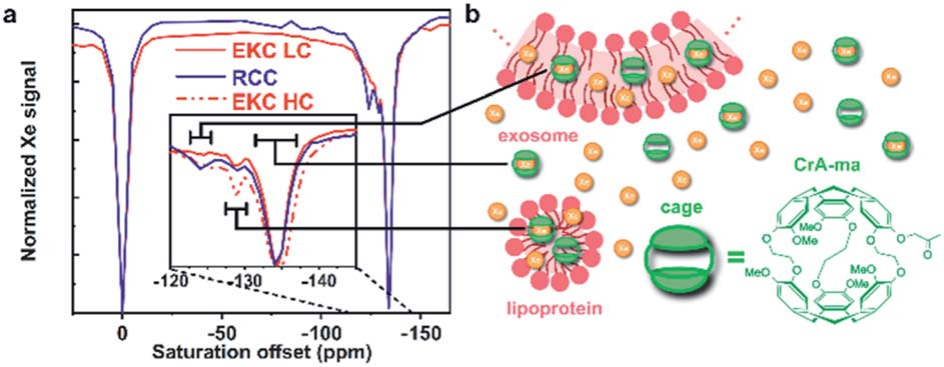
^129^Xe Hyper-CEST analysis of EVs. (a) Hyper-CEST spectra of EKC (with low and high concentrations, LC and HC, respectively) and RCC EVs. The black bars indicate the assigned range of the signals. (b) Illustrations of sample components in the ^129^Xe Hyper-CEST experiments of EVs. Xenon atoms may be free or encapsulated in CrA-ma cages in different locations: in bulk water or water encapsulated by exosomes or microvesicles; in lipid bilayer of larger particles (exosomes and microvesicles); and inside smaller particles (such as lipoproteins and exomers).

For the Hyper-CEST experiments, cryptophane-A mono-acid cages (CrA-ma) were introduced in the EV sample solutions to obtain a distinct Xe@CrA-ma in lipid signal. As illustrated in Fig. 4b, xenon atoms experience multiple potential environments in the sample: they can be free in the solution or encapsulated inside a CrA-ma cage in the solution; they can also stay inside the lipid bilayer or smaller aggregates such as lipoproteins or encapsulated in a CrA-ma cage in the lipid structures. The latter one is the most wanted type of signal in this context and it typically appears *ca.* 10 ppm downfield from Xe@CrA-ma in aqueous environment.^35^ The ^129^Xe Hyper-CEST spectra of the EKC and RCC exosome samples are shown in Fig. 4a. The spectra include a signal around 0 ppm arising from free Xe in solvent and in lipid structures; these signals are overlapping due to relatively fast exchange. However, the signals of encapsulated Xe provides resolution of different environments: the signal around −134 ppm corresponds to encapsulated Xe in aqueous environment, while the signals between −130 and −120 ppm originate from encapsulated Xe in cages in the lipid structures. The signals between −134 and −120 ppm were not observed in standard hyperpolarized ^129^Xe spectra; they became observable only due to the significant signal enhancement provided by the CEST mechanism.

There are two distinguished signals arising from the lipid structures with their maxima around −125 and −129 ppm. We hypothesize that they represent the larger (exosomes and microvesicles) and smaller (lipoproteins and exomers) nanoparticles observed in the DOSY and NTA analysis. The ^129^Xe Hyper-CEST analysis can also be considered as an additional validation of the bimodal or multimodal particle size distribution indicated by DOSY. The peak at −125 ppm is considerably narrower than the one at −129 ppm and thus indicates a presumably slower Xe exchange for the lipoprotein/exomer pool.

We fitted the high concentration EKC Hyper-CEST spectrum with a superposition of exponential Lorentzians as used in a two-pool model by Kunth *et al.*^60^ to get some quantitative insights. We assumed that the system includes three CEST pools and one detection pool, in accordance with Fig. 4. Furthermore, we assumed that the three CEST pools have predominantly direct exchange with the detection pool, but not between each other to a significant amount. The chemical shift difference between the CEST pools and free Xe is large and transverse relaxation rates in the CEST pools are assumed to be negligible as compared to the exchange rates and saturation transfer. This allows to obtain an upper limit for the exchange rates (we see no relevant indications to ascribe part of the observed signal widths to a shortened transverse relaxation in the CEST pools). The fitted spectrum is in a good agreement with the experimental data (see Fig. S4 in ESI†). According to the fitting results, the exchange rate of the EV pool characterized by the broad signal around −125 ppm is presumably much higher (about 833 s^−1^) than that of the lipoprotein/exomer pool characterized by the narrow signal around −129 ppm (presumably less than 10 s^−1^). Based on the current information, it is not possible to fully understand, why the exchange rates are so different for these two pools. One conceivable explanation could be that the concentration of non-encapsulated Xe around the cages in the lipid bilayer of EVs is much higher than in non-membranous exomers and lipoprotein, thus providing enough Xe on the immediate vicinity to enable faster exchange. According to the fit, the amount of Xe bound to the pool was about 0.24% for both EV and lipoprotein/exomer pools.

### Immuno-electron microscopy, western blot and bioanalyzer traces of RNA

**Fig. 5:**
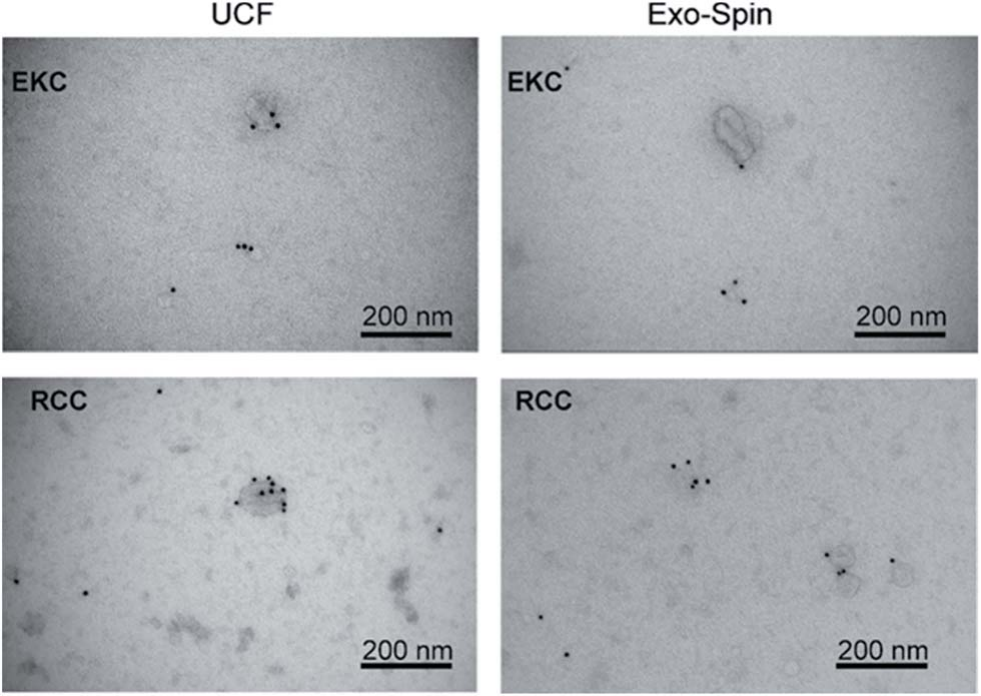
TEM images of EKC and RCC samples after negative staining with uranyl acetate and immunostaining with anti-CD63 antibody.

We analyzed expression of typical EV markers in the samples used for NMR measurements (see Supplementary materials and methods in ESI†). TEM with immunogold labelling (Fig. 5) demonstrated that the majority of EVs in the 30–200 nm size range were positive for CD63, a widely used exosomal biomarker.^51^ Only the smaller particles, more numerous in RCC-derived EVs samples as compared to EKC, were mostly CD63-negative. In contrast to RCC and EKC, only a few milk EVs expressed CD63 (Fig. S3 in ESI†).

**Fig. 6:**
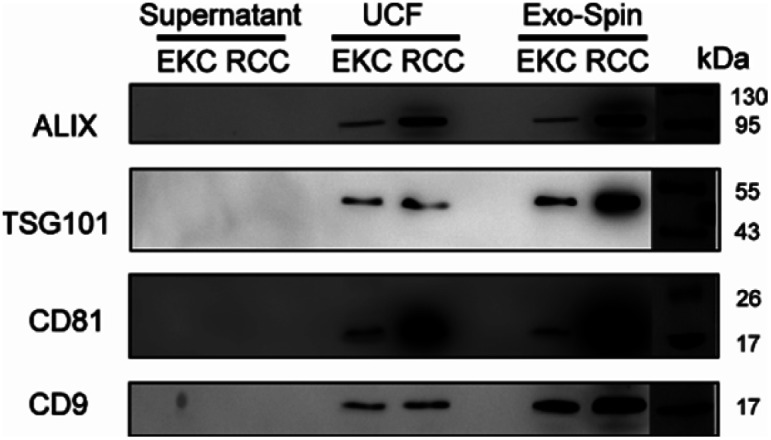
Western blot of EKC and RCC EV samples, isolated by sequential ultracentrifugation (UCF) or by using Exo-spin columns (see Materials and methods), with antibodies against common EV markers ALIX, TSG101, CD81, and CD9. EV-depleted supernatants (after 100 000*g* centrifugation) were used as controls. Protein marker (PageRuler™ Prestained Protein Ladder, Thermo Fisher, 26616) is shown to the right.

For a more detailed analysis of the markers expression we performed western blots of EV samples with antibodies against ALIX, TSG101, CD81, and CD9 (Fig. 6). All these proteins were described as typical components of exosomes and EVs in general, being either transmembrane proteins (CD81 and CD9) or components of internal EV cargo (ALIX and TSG101).^51^ An equal amount of proteins was loaded for each sample (Fig. S5 in ESI†). Argonaute2 (Ago2), which binds to free miRNA in serum,^52^ served as a negative control (Fig. S6 in ESI†). All the assayed markers were detected in higher amounts in EVs isolated from RCCs with the use of ultracentrifugation followed by Exo-Spin columns in comparison with simple ultracentrifugation (UCF). Exo-spin based purification led to enrichment of CD9- and TSG101-positive EVs (but not of ALIX- and CD81-positive ones) in the samples from EKC. Negative control samples (cell culture supernatants after 100 000*g* centrifugation) did not contain any detectable amounts of EV markers.

EVs isolated from milk by using UCF with subsequent filtration contained exosomal markers ALIX, TSG101 and CD81 (Fig. S8†). In milk EVs, isolated by ultracentrifugation alone, CD81 and TSG101 were less expressed and ALIX not detectable. Exosomal markers (except for trace amounts of TSG101) were not visible in control samples (milk before EV isolation). Bioanalyzer traces of RNA from EV samples correspond to the pattern typical of EV RNA (Fig. S8†).

The strengths of the DOSY analysis described in this article are its non-invasiveness, as it does not require any labelling, and its ability to detect very broad particle size distribution. The weakness of the method is low sensitivity. This led to a long experiment time of 50 hours due to the need for signal averaging (1024 scans accumulated). The sensitivity could be increased by using a higher field NMR instruments, larger concentrations of particles and larger amounts of sample. Another potential approach to improve the sensitivity is to use more efficient methods to collect diffusion data.^53,54^

For our proof-of-concept study, we tested several EV isolation methods: sequential ultracentrifugation, filtration, and size exclusion chromatography with Exo-spin™ columns. Because of low sensitivity of DOSY analysis, we needed to maximize EV yields; therefore OptiPrep™ density gradient centrifugation, that we previously described as a method to isolate exosomes from EKC-derived cell cultures,^55^ was not used in the present study.

The membrane material is clearly “labelled” by the self-embedding, hydrophobic CrA-ma and this improves the spectral dispersion for Xe inside the lipid phase. The chemical shift resolution for uncaged Xe is pretty similar between aqueous and membrane phase^56^ and shows strong overlap in lipid suspension systems.^34^ Introducing CrA-ma as a membrane label consistently produced two Xe@CrA-ma Hyper-CEST signals in previous studies for synthetic systems^34,35,57^ as well as in cell studies.^58,59^ A covalently phospholipid-anchored CrA-ma cage also confirmed this observation.^57^ Together with the assumption that most CrA-ma resides in the aqueous phase due to the small fraction of the lipid phase, we thus take the dominant CEST peak at −134 ppm as a reference and assign the signal at −124 ppm to the EV membrane environment as this comes closest to the conditions in previous studies.^35,61^

The additional signal at −129 ppm has not been reported before. As it is in between the chemical shifts for Xe@CrA-ma in aqueous and EV environment, it seems reasonable to assign this to cages in smaller aggregates such as lipoproteins as such an environment would facilitate faster exchange of the Xe host in and out of the water pool. This would push the chemical shift towards that of the dominant signal. Increasing the concentration of the EKC sample clearly increased the CEST responses and thus confirm the interpretation that these peaks are associated with the lipid environments.

## Conclusions

We demonstrated the feasibility and usefulness of two different NMR techniques in the characterization of EV samples. ^1^H DOSY analysis provides the opportunity to determine size distribution of nanoparticles in the samples noninvasively, without chemical labelling. The method can detect a very broad range of particle sizes, from sub-nanometer to hundreds of nanometers, including small nanoparticles (below 70 nm), which are invisible in the standard NTA but visible in the TEM images. Therefore, the method provides means to determine the purity of isolated EV samples.

The analysis revealed clear differences between the samples isolated from different sources; for example, the average particle size in the milk samples was much larger than in the EKC and RCC samples. The differences between EVs produced by two cell types were less prominent. The ^129^Xe Hyper-CEST technique provided discrimination of sample components in a way that is not available in standard ^129^Xe NMR spectra. It supports the existence of the multimodal particle size distribution observed by DOSY and TEM. Western blot analysis and immuno-electron microscopy confirmed expression of exosomal markers such as ALIX, TSG101, CD81, CD9, and CD63 in the EV samples. The sensitivity and information content of the DOSY and Hyper-CEST analysis can be improved significantly by optimizing and standardizing the sample preparation, its concentrations and volumes, and by using higher magnetic fields and more efficient detection techniques.^53,54^ Furthermore, the Hyper-CEST method could be made more specific by functionalizing cages with antibodies against exosomal surface markers, *e.g.* like translating the approach that has been done for CD14 to exosome-related targets such as CD81 or CD63.^59^ Therefore, the techniques have a potential to become very valuable tools in the analysis of exosomes and other biological nanoparticles.

## Author contributions

M. S. U.: carrying out the DOSY NMR and Hyper-CEST experiments, analysis of the results, writing the first version of the manuscript. V. V. Z.: planning, implementation and carrying out of the DOSY NMR experiments and analysis of the results. A. S.: planning the EV research, EV sample preparation, manuscript writing and analysis of the results. A. Z.: exosome sample preparation, NTA and western blot. Sirja Viitala: purification of the milk exosome samples. Santeri Kankaanpää: western blot of milk samples, RNA extraction and analysis. Sanna Komulainen: supervision of initial NMR experiments. L. S.: planning, implementation and carrying out of Hyper-CEST experiments and analysis of the results. Seppo Vainio: planning the exosome research and analysis of the results. V.-V. T.: planning the NMR experiments and exosome research, writing the polished version of the manuscript. All the authors contributed on the final version of the manuscript.

## Conflicts of interest

There are no conflicts to declare.

## Supplementary Material

SC-012-D1SC01402A-s001
