# Supplementary material for: Identification of extracellular nanoparticle subsets by nuclear magnetic resonance
Source: Chem Sci. 2021 Apr 29;12(24):8311–9. doi: 10.1039/d1sc01402a (PMC8221169; doi:10.1039/d1sc01402a)
Supplement: SC-012-D1SC01402A-s001 [file SC-012-D1SC01402A-s001.pdf]

Electronic Supplementary Material (ESI) for Chemical Science.
This journal is © The Royal Society of Chemistry 2021

Electronic Supporting Information (ESI) to the article

Identification of extracellular nanoparticle subsets by nuclear magnetic resonance

Md Sharif Ullah,[a] Vladimir V. Zhivonitko,[a]* Anatoliy Samoylenko,[b]* Artem Zhyvolozhnyi,[b] Sirja Viitala,[c] Santeri Kankaanpää,[c] Sanna Komulainen,[a] Leif Schröder,[d,e]* Seppo J. Vainio[b] and Ville-Veikko Telkki[a]*

[a]*NMR Research Unit, University of Oulu, Finland*
[b]*Laboratory of Developmental Biology, Infotech Oulu, Oulu Center for Cell-Matrix Research, Kvantum Institute and Faculty of Biochemistry and Molecular Medicine, Oulu, Finland*
[c]*Natural Resources Institute Finland (Luke), Production Systems, Jokioinen, Finland*
[d]*Leibniz-Forschungsinstitut für Molekulare Pharmakologie (FMP), Molecular Imaging, Berlin, Germany*
[e]*Division of Translational Molecular Imaging, German Cancer Research Center (DKFZ), Heidelberg, Germany*

Supplementary materials and methods

NTA and immuno-electron microscopy

Initially, the concentrations and size distributions of the EV samples were characterized by NTA using the Malvern Panalytical NanoSight NM300 instrument equipped with a 405 nm laser. According to NTA, the concentrations varied between 10^{10} and 10^{12} particles/ml (see Table S1 in ESI†). Some EV samples were also analyzed by transmission electron microscopy (TEM). 2 µl of each sample were deposited on a Formvar carbonated grid (glow-discharged) and after negative staining with 2% uranyl acetate and immunostaining with anti-CD63 antibody (LAMP-3, MBL, Nagoya, Japan) examined using the Tecnai G2 Spirit transmission electron microscope (FEI, Eindhoven, The Netherlands). Protein A-gold complex (10 nm) served to detect the primary anti-CD63 antibody. Images were captured with a charge-coupled device camera (Quemesa, Olympus Soft Imaging Solutions GMBH, Münster, Germany).

Western blot

Milk-derived EVs (10 µg protein per sample) were prepared in Laemmli sample buffer (138 mM Tris-HCl pH 6.8 (Sigma Aldrich), 6 M Urea (Sigma Aldrich), 4.3% w/v SDS (Merck) 22% w/v glycerol (Merck), 5% v/v β-mercaptoethanol (Sigma Aldrich) and bromophenol blue (Merck)) and incubated for 30 minutes at 37C. Samples were centrifuged for 5 min at 14 000 g prior to loading to the gel. Protein separation was done using 12% polyacrylamide gel (0.192 M Tris-HCl pH 8.8 (Sigma Aldrich), 3.35 M urea (Sigma Aldrich), 0.22% SDS w/v (Merck), 12% w/v acrylamide (19:1 acrylamide/bis-acrylamide 40% solution BioRad), 0.04 % v/v tetraethyl methyl diamine (TEMED, GE healthcare) and 0.025 % w/v ammonium persulfate (Amersham Biosciences)) with running buffer (192 mM Glycine, 25 mM Tris, 3.5 mM SDS). Electrophoresis was run with BioRad Tetra-cell electrophoresis dock with current of 15 mA/gel and voltage of 170 V. Proteins were transferred

using SemiDry transfer unit (BioRad) with 25 V and 1 mA/cm3 as transfer conditions. Tris-Glycine buffer (2.5 mM Tris, 19.2 mM glycine, BioRad) with 20 % methanol was used as transfer buffer.

Membranes were blocked using 5% fish gelatin (Thermo fisher) in TBS (Trizma buffered saline) for 1 h prior of incubation with primary antibodies: rabbit anti-Alix (E6P9B, Cell signaling technology; 1:2500), mouse anti-CD81 (sc-166029, Santa Cruz biotechnologies; 1:1000), rabbit anti-TSG101 (Nordic BioSite, ABB-709, rabbit polyclonal; 1:2500) overnight with mild rocking at 4°C in TTBS with 1 % fish gelatin. Secondary anti-rabbit (AS 09602, Agrisera (Sweden)) and anti-mouse (Sc-516102, Santa Cruz biotechnologies) antibodies were incubated in 1:5000 dilution at RT with mild rocking in TTBS for 2 h. Membranes were washed twice with TTBS and once with TBS for 5 minutes prior of developing with ECL (0.1 M Tris HCl pH 8.5, 0.198 mM P-coumaric acid (Sigma), 1.25 mM Luminol (Sigma) and 0.9 % HOOH (Merck)) and imaged with ChemidocMP (BioRad).

EVs isolated from RCC and EKC culture media were lyzed in RIPA buffer (Cell Signaling Technology) containing protease inhibitor cocktail cOmplete™ ULTRA (Roche) and phosphatase inhibitor cocktail 2 (Sigma-Aldrich). Proteins (10 µg per sample) were separated on 10% SDS PAGE gel, and then transferred to nitrocellulose membrane. Antibodies against exosomal markers CD81 (Santa Cruz Biotechnology, sc-166029; 1:1000), CD9 (Abcam, ab92726; 1:1000), ALIX (Abcam, ab117600; 1:1000), and TSG101 (Santa Cruz Biotechnology, sc-7964; 1:1000), as well as anti-Argonaute-2 antibody (Abcam, ab32381) were incubated overnight at 4°C with the membranes, and washed several times in PBST buffer. The respective secondary peroxidase-conjugated IgG antibodies (Invitrogen, 1:5000) were then applied to the membranes. The Lumi-Light Western Blotting Substrate (Roche Diagnostics, Switzerland) was used to visualize the bound antibodies.

RNA isolation from EVs

RNA from milk samples was extracted using a modified Total exosome RNA isolation (Invitrogen) protocol. Samples were suspended in PBS overnight on slow rocking at + 4˚C. Samples were mixed with the lysis buffer and incubated on ice for 5 minutes. Equal volume of acid-phenol-chloroform mixture was added and mixed by slowly inverting for 3 minutes. After incubation samples were centrifuged for 5 minutes with maximum speed with Eppendorf centrifuge 5415 D. Upper phase was extracted and mixed with 1.25 times the volume of 100 % ethanol. Samples were centrifuged through purification filter cartridges and washed three times in total with wash solution 1 and 2/3. Samples were eluted with 2 times 50 µl of RNAse free water. Quality was analyzed with Bioanalyzer 2000 RNA 6000 Pico kit (Agilent) and quantity was analyzed with Qubit 4 and microRNA assay (Thermo Scientific).

For RNA isolation from cell-derived EVs 600 µl of Qiazol (Qiagen) was added to 100 µl of EVs and mixed by vortexing. After 2-5 minutes incubation at RT 90 µl chloroform was added, then extraction of RNA was done

according to ExRNeasy (Qiagen) protocol using minElute RNA column. RNA was eluted in 14 µl water, 1 ul was used for profiling with bioanalyzer pico 6000 chips using total eukaryotic RNA program.

Supplementary results and discussion

DOSY and NTA particle size distributions

Table S1. EV samples studied in this work as well as data of the DOSY NMR and NTA analysis.

Sample type	Sample name	Isolation method	Sample concentration Particles/ml; NTA	Range of b values in DOSY ($\times 10^9$, s/m^2)
Milk	Milk 1	Filtration	1.45×10^{12}	3.85-965.10
	Milk 2	Sequential ultra-centrifugation	1.83×10^{11}	3.85-965.10
Embryonic kidney cell (EKC)	EKC 1	Centrifugation plus Exo-Spin	7.25×10^{10}	3.85-965.10
	EKC 2	Centrifugation plus Exo-Spin	1.04×10^{12}	3.85-965.10
	EKC 3	Centrifugation plus Exo-Spin	1.04×10^{12}	3.85-323.50
	EKC 4	Centrifugation plus Exo-Spin	5.76×10^{11}	3.85-965.10
	EKC 5	Sequential ultracentrifugation	7.47×10^{10}	3.85-523.98
Renal carcinoma cell (RCC)	RCC 1	Centrifugation plus Exo-Spin	4.42×10^{11}	3.85-965.10
	RCC 2	Centrifugation plus Exo-Spin	7.95×10^{10}	3.85-323.50
	RCC 3	Centrifugation plus Exo-Spin	2.54×10^{11}	3.85-523.10
	RCC 4	Sequential ultra-centrifugation	6.47×10^{11}	3.85-267.34

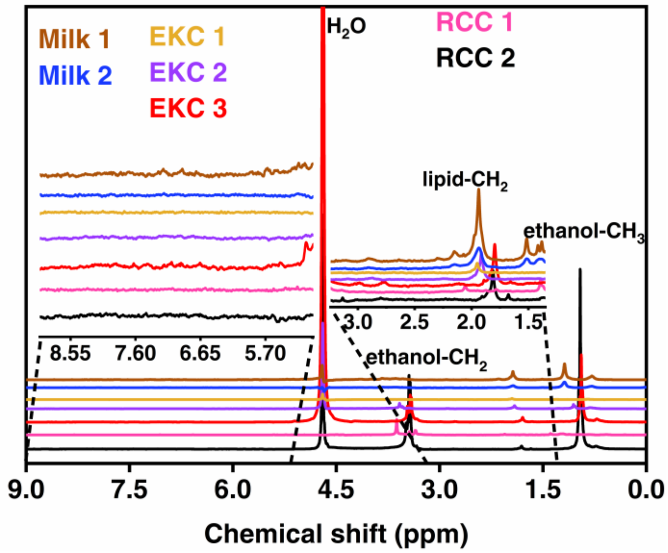

Fig. S1 ^{1}H diffusion-weighted NMR spectra of EV samples from the different sources (milk, EKC, RCC). The signals from exosomes are visible in the range of 1.8-2.0 ppm. There are no signals visible within 5.0-9.0 ppm.

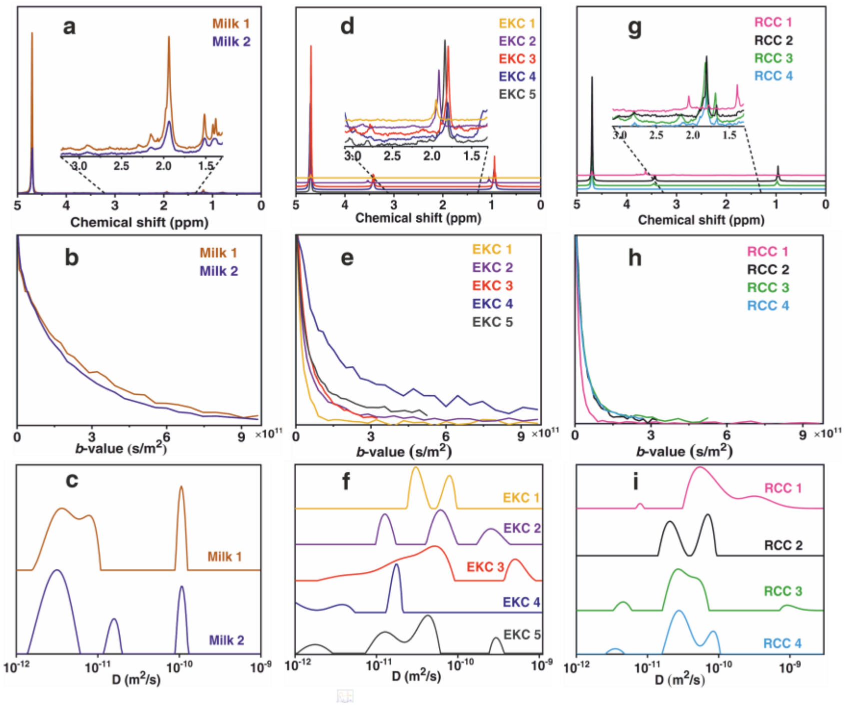

Fig. S2 ^{1}H diffusion-weighted NMR spectra of milk (a), EKC (d) and RCC (g) EV samples. (b), (e) and (h): Corresponding diffusion decay curves. (c), (f) and (i): Corresponding diffusion coefficient. The diffusion coefficients were converted into particle size distributions by using the Stokes-Einstein equation and dynamic viscosity of H_2O of $1{\cdot}10^{-3}$ Pa·s at T = 293 K.

TEM images

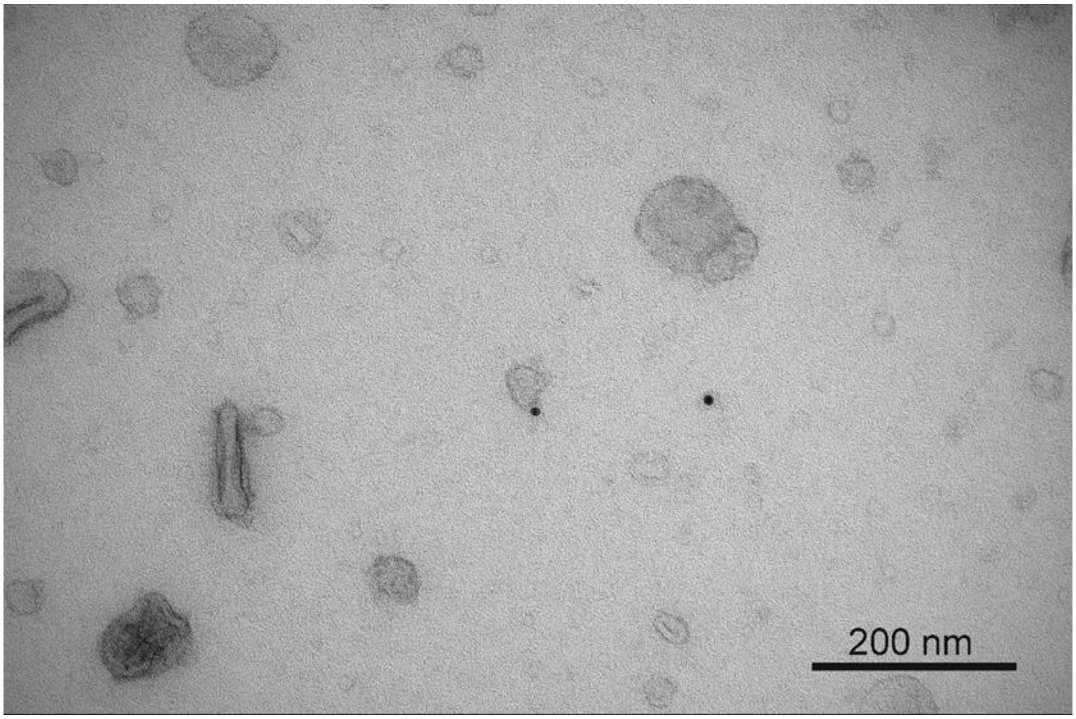

Fig. S3 TEM images of a milk sample after negative staining with uranyl acetate and immunostaining with anti-CD63 antibody.

^{129}Xe Hyper-CEST analysis

Given the assumptions described in Kunth *et al.*,[1] the spectrum with the best resolution and high signal stability could be used for fitting three exponential Lorentzians to the data according to

$$S(\Delta\omega) = \max\left(S_0 e^{-t_{\mathrm{sat}}\left(\sum_i \mathcal{L}_i(\Delta\omega)\right)}; R\right)$$

with a fixed saturation time t_{sat} = 25 s and the Lorentzian profiles (i = 1-3)

$$\mathcal{L}_i(\Delta\omega) = \frac{\lambda_i \left(\frac{\Gamma_i}{2}\right)^2}{\left(\frac{\Gamma_i}{2}\right)^2 + (\Delta\omega - \delta_i)^2}.$$

δ_i is the chemical shift of the CEST response, Γ_i the full width at half maximum, and λ_i the depolarization rate for on-resonant saturation ($\Delta\omega = \delta_i$). R represents a Rician noise floor that originates from integrating the Xe spectra of dissolved Xe and yields values ≠ 0. Γ_i is determined by the applied saturation power γB_1 and the exchange rate $k_{\mathrm{BA},i}$ from each CEST pool (B$_i$) into the free solution pool (A): $\Gamma_i = 2\sqrt{(\gamma B_1)^2 + \left(k_{\mathrm{BA},i}\right)^2}$. This assumes that no significant transverse relaxation rate contributes to the width of the CEST responses. This condition is usually at least known for the CEST signal of CrA-ma in water. Then, the depolarization rate $\lambda_i \approx f_{\mathrm{B},i} k_{\mathrm{BA}} \frac{(\gamma B_1)^2}{\left(k_{\mathrm{BA},i}\right)^2 + (\gamma B_1)^2}$ can be used to obtain the fraction of bound Xe in each pool, $f_{\mathrm{B},i}$, (relative to the pool of free Xe) after the exchange rate has been determined from the width Γ_i.

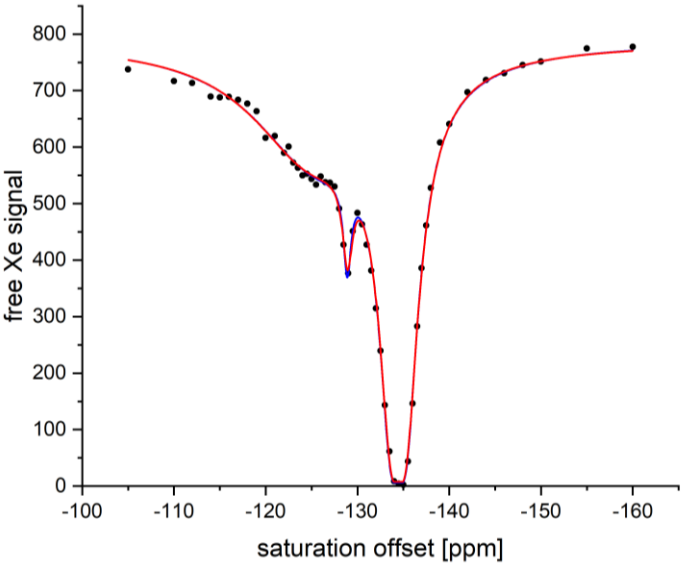

Fig. S4 Fitted Hyper-CEST data of the high concentration EKC sample. Black dots: experimental data without normalization to the starting magnetization. Red curve: fitted spectrum with the minimum line width Γ_3 = 1.22 ppm as prior knowledge. Blue curve: fitted spectrum with free parameters.

The fit does not require to normalize the data and leaves the starting magnetization S_0 as free parameter. The CEST response from excess CrA in solution is represented by $\mathcal{L}_1$, while $\mathcal{L}_2$ and $\mathcal{L}_3$ describe the responses from CrA in larger lipid vesicles (previously described signal with ca. 10 ppm downfield shift) and the newly discovered signal from CrA in the lipoprotein/exomer environment.

The applied saturation power of 2.5 mW corresponds to γB_1= 5.69 µT and should yield a minimum line width of Γ_i ≈ 1.22 ppm. A completely free fit yields an underestimated Γ_3= 0.94 ppm (blue fitting line). This value was thus restricted to at least 1.22 ppm and the fitting was repeated (red spectrum). Fitting results are shown in Table S2.

Table S2. Hyper-CEST fitting results for the high concentration EKC sample

parameter	fit result
S_0 [a.u.]	786.4 ± 0.2
t_{sat} [s]	25 ± 0
δ_1 [ppm]	-134.453 ± 0.002
Γ_1 [ppm]	1.750 ± 0.009
λ_1 [s^{-1}]	0.243 ± 0.002
δ_2 [ppm]	-125.227 ± 0.035
Γ_2 [ppm]	15.103 ± 0.068
λ_2 [s^{-1}]	0.01189 ± 0.00003
δ_3 [ppm]	-128.915 ± 0.004
Γ_3 [ppm]	1.220 ± 0.013
λ_3 [s^{-1}]	0.01334 ± 0.00009
R [a.u.]	7.59 ± 0.95
χ^2	4.53149
R^2 (COD)	0.99983
corr. R^2	0.99983

Immuno-electron microscopy, Western blot and Bioanalyzer traces of RNA

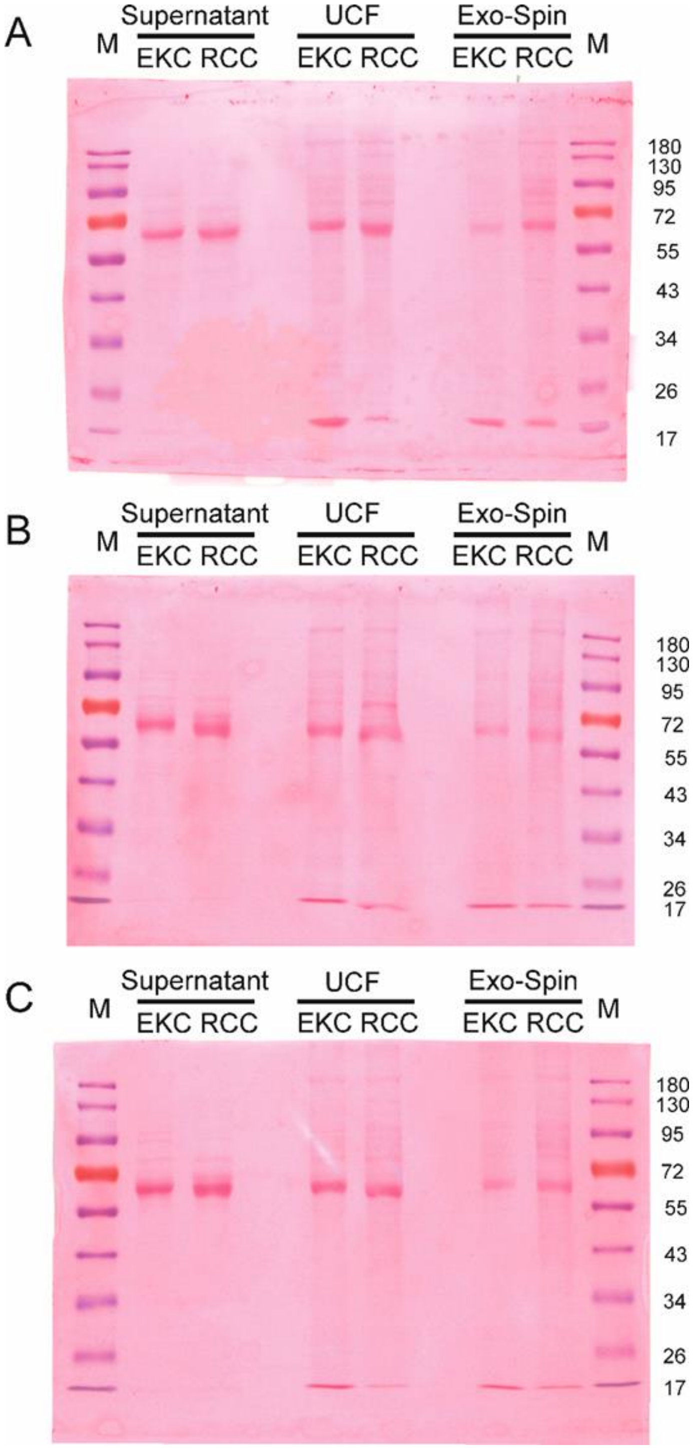

Fig. S5 Ponceau S staining of membranes used for Western blotting with Alix, TSG101 (both A), CD81 (B), and CD9 (C) (corresponding blots are shown in Figure 6). M – protein ladder (marker).

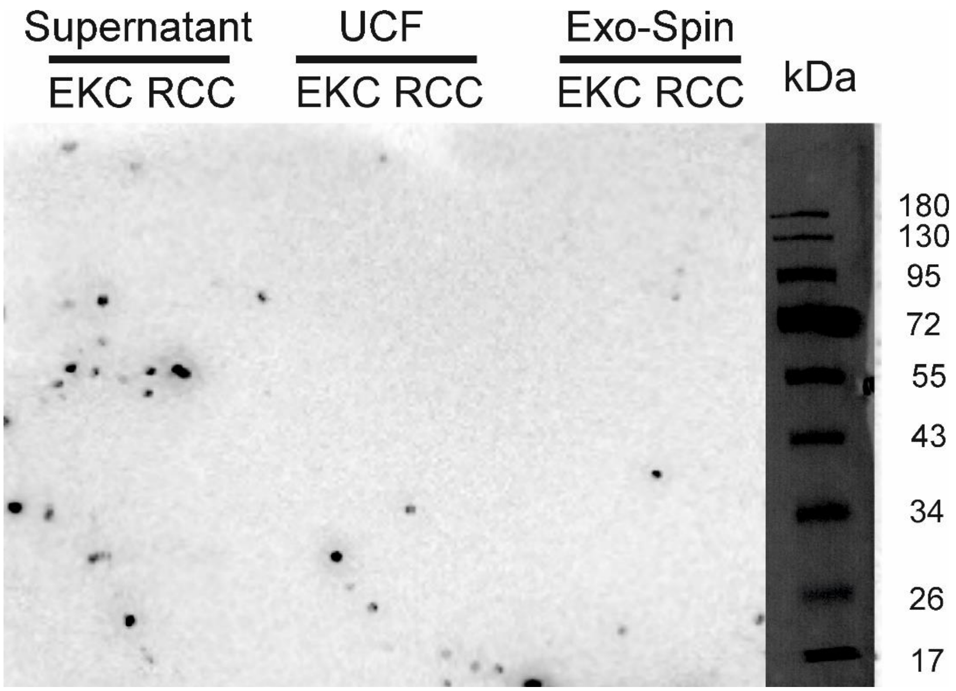

Fig. S6 Western blot of EKC and RCC EV samples, isolated by sequential ultracentrifugation (UCF) or by using Exo-spin columns (see Materials and methods), with antibodies against Argonaute-2. Protein marker (PageRuler™ Prestained Protein Ladder, Thermo Fisher, 26616) is shown to the right.

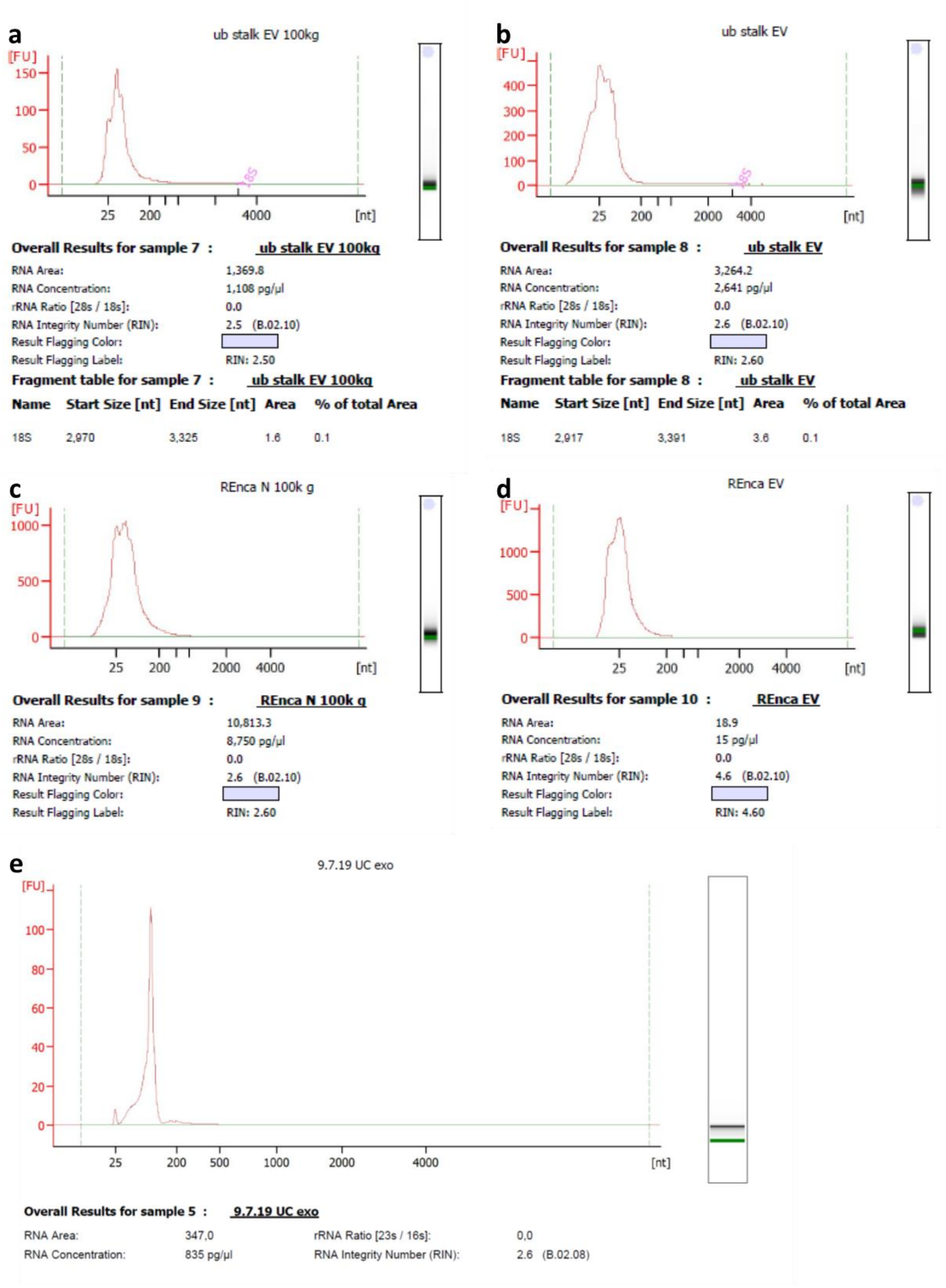

Fig. S7 Characterization of RNA isolated from EV samples: (a) EKC after ultracentrifugation; (b) EKC after Exo-spin; (c) RCC after ultracentrifugation; (d) RCC after Exo-spin; (e) Milk after ultracentrifugation. For each sample 1 µl of isolated RNA was used for profiling with Bioanalyzer pico 6000 chips.

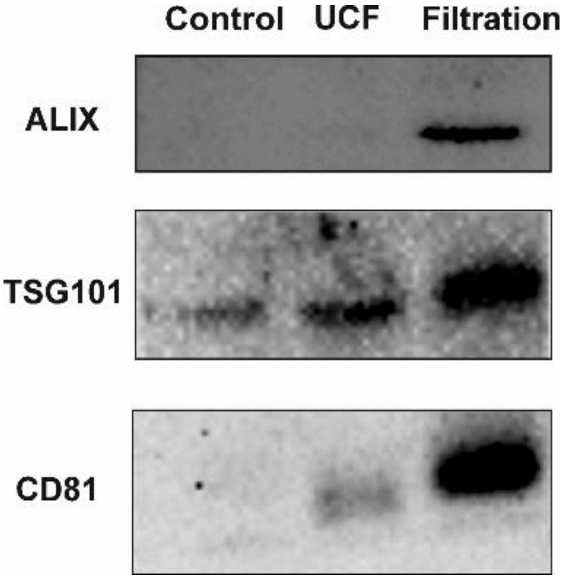

Fig. S8 Western blot of milk EV samples, isolated by sequential ultracentrifugation (UCF) or by using filtration (see Materials and methods), with antibodies against common EV markers ALIX, TSG101, and CD81. Milk samples before EV isolation were used as controls.

References

1 M. Kunth, C. Witte, L. Schröder, *NMR Biomed.*, 2015, **28**, 601-606.
